# Concordance between reflectance confocal microscopy and histopathology for the diagnosis of acral lentiginous melanoma

**DOI:** 10.1111/srt.13570

**Published:** 2024-01-10

**Authors:** Yunmin Zou, Xiaohong Zhu, Rushan Xia

**Affiliations:** ^1^ Department of Dermatology Wuxi No.2 People's Hospital Wuxi Jiangsu Province China

**Keywords:** acra, histopathology, melanoma, reflectance confocal microscopy

## Abstract

**Background:**

Acral lentiginous melanoma (ALM) is a highly malignant and invasive type of melanoma with unique locations of onset. Its incidence is increasing and early diagnosis is challenging. Reflectance confocal microscopy (RCM) is a non‐invasive technique that provides an accurate image of tissue pathology. There are few reports on the use of RCM for the assessment of ALM.

**Materials and methods:**

In this retrospective study, data from 31 patients with a clinical diagnosis of ALM were collected. RCM image features were compared with histopathological findings to determine the concordance between the two methods. The sensitivity, specificity, positive predictive value, and negative predictive value of RCM for the diagnosis of ALM were evaluated.

**Results:**

RCM and histopathology findings were concordant in 29 of 31 patients (93.5%). There were no false‐negative results, although there were two false positives in RCM diagnosis. The sensitivity of RCM for diagnosing ALM was 100%, specificity was 50%, positive predictive value was 93.1%, and negative predictive value was 100%.

**Conclusions:**

RCM showed substantial concordance with histopathology in the diagnosis of ALM. It is a reliable and valuable non‐invasive diagnostic tool that holds promise for the early diagnosis of ALM.

## 1 INTRODUCTION

The incidence of malignant melanoma (MM) has increased in recent years, with an annual growth rate of 3%−5%. MM is a highly malignant tumor with a poor prognosis. Acral lentiginous melanoma (ALM), which is the predominant subtype in China, is characterized by high malignancy and aggressiveness, underscoring the importance of early detection. Early‐stage ALM is difficult to diagnose because of the absence of typical MM clinical features and the unique sites of onset. Biopsy samples are frequently limited in quantity and typically have a sparse presence of tumor cells, posing a challenge to histopathological diagnosis. These factors contribute to the misdiagnosis or delayed identification of ALM, which limits the options for providing the most effective treatment. Reflectance confocal microscopy (RCM) is a non‐invasive, in vivo imaging technology that allows the visualization of skin cell‐level changes, providing images that closely resemble histopathology findings. In a recent meta‐analysis,^1^ RCM showed a sensitivity of 92% and a specificity of 70% for the diagnosis of MM, demonstrating a superior diagnostic performance to that of dermoscopy. There are several reports describing the use of RCM for the diagnosis of non‐ALM melanoma; however, studies of RCM in patients with ALM are limited. In this study, we retrospectively analyzed the RCM characteristics of ALM and assessed the concordance between RCM and histopathology. The present results provide information that may help the early diagnosis of ALM.

## 2 MATERIALS AND METHODS

Thirty‐one patients who were clinically diagnosed with ALM in Wuxi Second People's Hospital between January 2018 and December 2022 were included in the study; the study cohort comprised 14 men and 17 women aged 44−86 (63.8 ± 12.68) years. All patients underwent RCM examination, and high‐quality RCM images and definite pathological diagnostic results were obtained. This study was approved by the Ethics Committee of Wuxi Second People's Hospital.

The instruments used in the study were the VIVASCOPE 1500 reflectance confocal imaging system (Lucid Technology) and non‐invasive real‐time pathological analysis system (Conbio, China). An appropriate body position was selected according to the patient's skin lesions, and the affected areas were identified. Distilled water and an ultrasound coupling agent were used as the medium. An 830 nm near‐infrared laser beam was used as the light source with an output power ranging from 0 to 22 mW. The lateral resolution was 1.25 μm, and the axial resolution was 5 μm with a 30× water immersion objective lens. The criteria for the diagnosis of MM were described previously.^2^, ^3^ The two primary criteria were atypical cells (nests) at the dermal‐epidermal junction, and disordered epidermal structures along with the disappearance of papillary rings. Four secondary criteria were intraepidermal dendritic and spindle cells, scattered or widespread presence of round or oval Paget‐like cells, nests of anomalous tumor cells, and nucleated cells in the dermal papilla. ALM was diagnosed if two primary criteria or one primary criterion plus two secondary criteria were met. The results were interpreted by two physicians with experience in RCM, and histopathological examination was performed at the same site. The concordance between the two methods was analyzed, and the sensitivity, specificity, positive predictive value, and negative predictive value of RCM for the diagnosis of ALM were calculated.

## 3 RESULTS

**FIGURE 1:**
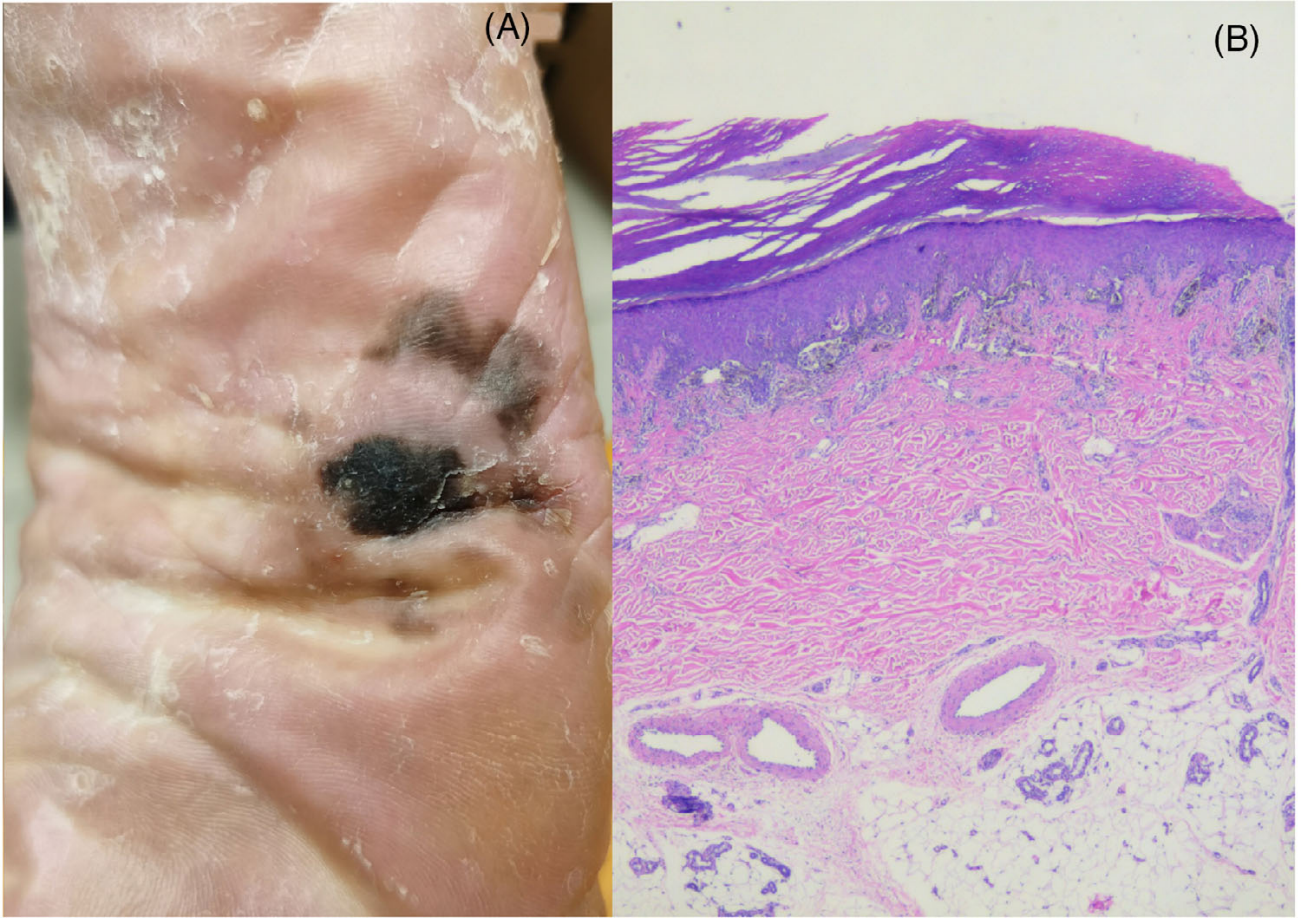
Clinical manifestations and histopathology of ALM. (A) ALM is manifested as irregular dark brown and light brown patches. (B) Histopathological features of ALM: Melanocytes move up into the epidermis and spread in a Paget manner. Tumor cells are observed at the dermal‐epidermal junction and in the dermis. Cells are scattered or distributed in nests, and show different sizes and irregular or abnormal shape (HE, ×100).

Among the 31 patients, the location of lesions was the palm in two cases, fingers in six cases (with five involving the nails), the soles of the feet in 13 cases, the heels in four cases, the dorsum of the foot in two cases, and the toes in four cases (all involving the toenails). Skin lesions appeared as dyspigmented, irregularly bordered brown or dark brown patches or plaques (Figure 1A). Some extended onto the nail plate, and some exhibited surface ulceration. The diameter of the skin lesions was 0.5−4.6 cm (average diameter, 2.8 cm), and the duration of the disease ranged from 1 to 40 years (average, 5.2 years).

**FIGURE 2:**
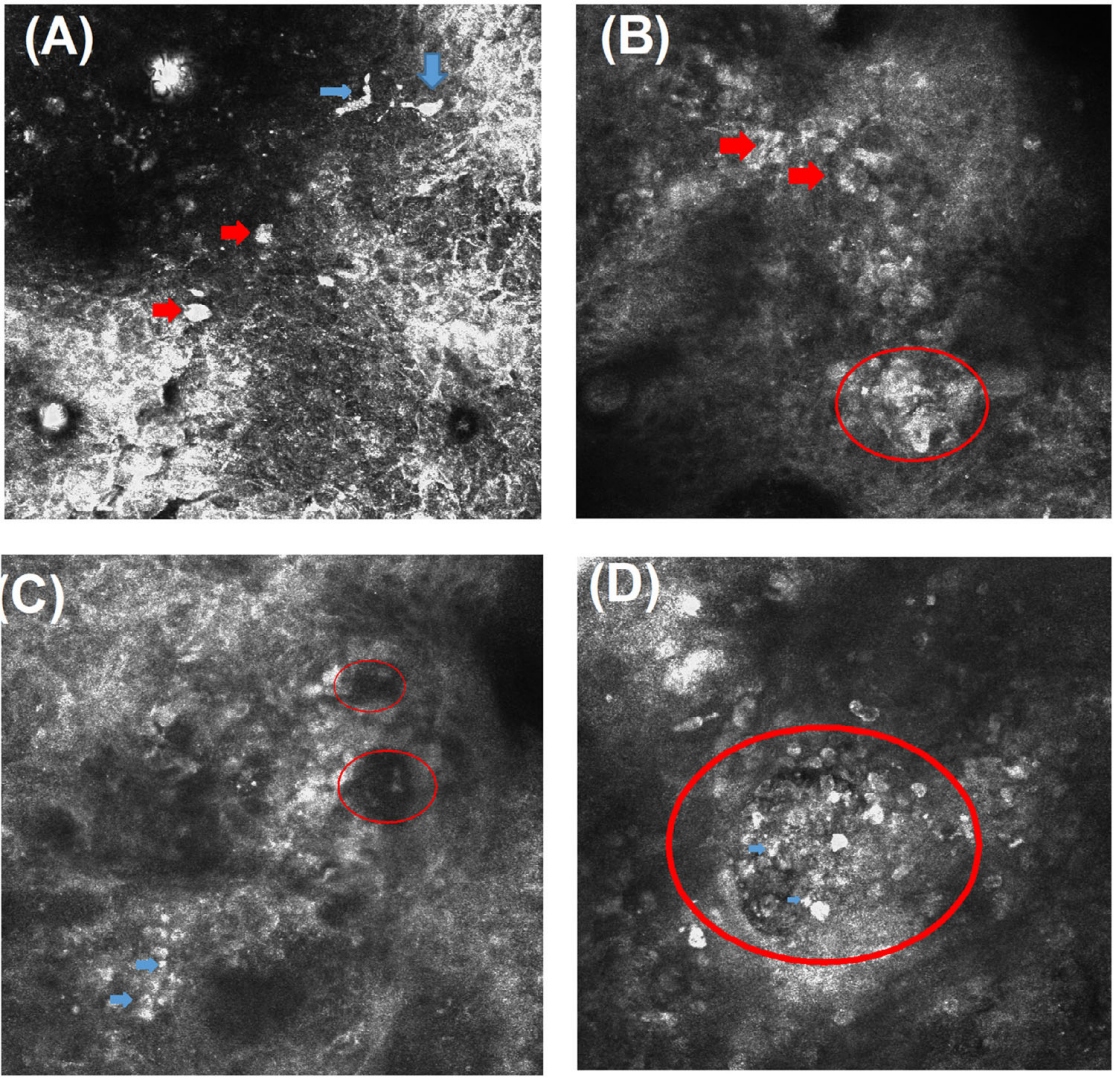
RCM characteristics of ALM. (A) Round (red arrow) and dendritic cells (yellow arrow) are scattered in the superficial stratum corneum of the epidermis. (B) Round and oval Paget‐like cells (red arrow) are distributed in nests in the epidermis (red circle). (C) Irregular highly refractive cells and disordered arrangement at the dermal‐epidermal junction; disappearance of the papillary ring is observed (red circle), as well as scattered highly refractive granules (blue arrow). (D) Nucleated cells (red circle) and highly refractive granules (blue arrow) in the dermal papilla.

Typical RCM features are shown in Figure 2 and summarized in Table 1. Of the 31 cases, 30 exhibited disordered epidermal structures and disappearance of honeycomb structures. Scattered spindle and dendritic cells (Figure 2A) were present in the epidermis in 18 cases, whereas round and oval Paget cells (Figure 2B) were observed in 6 cases. A mixture of these cell types was observed in three cases, whereas triangular cells were present in five cases. Uneven highly refractive granules were observed in 18 cases (Figure 2C). Twenty‐two cases had disordered epidermal structures at the junction of the dermis and epidermis accompanied by the disappearance of papillary rings (Figure 2C), and 20 cases had nested and abnormally shaped highly refractive cells with visible dendritic protrusions and an enhanced refractive index. One case had gyrus‐like structures and highly refractive particles in the epidermis, and three cases showed scattered, large, bright nucleated cells in the dermal papilla (Figure 2D). In two cases, scattered highly refractive particles were observed. In one case, tumor cell nests that appeared as nodules arranged in a palisade shape were detected in the dermis, and low‐refractive fissures were observed surrounding the tumor. Among the 31 cases, 29 were diagnosed as ALM, 1 was diagnosed as seborrheic keratosis, and 1 was diagnosed as basal cell carcinoma.

**TABLE 1:** Correspondence between RCM characteristics and histopathology in the 27 ALM cases.

Lesion layer	RCM	Histopathology
Epidermis	Disordered epidermal arrangement (*n* = 27, 100%)	Disordered epidermal arrangement (*n* = 27, 100%)
	Spindle, dendritic, round or oval cells (*n* = 21, 77.8%)	Upward transition of melanocytes with varying sizes and irregular shape (*n* = 20, 74.1%)
	Uneven high‐refractive granules (*n* = 18, 66.7%)	Melanin granules (*n* = 16, 59.3%)
Epidermal‐dermal junction	Disordered epidermal structure and disappearance of the papillary ring (*n* = 22, 81.5%)	Scattered or nest‐arranged melanocytes (*n* = 27, 100%)
	Nested atypical high‐refractive cells (*n* = 20, 74.1%)	Vary‐sized cells (nests) with darkly stained atypical nuclei (*n* = 27, 100%)
Dermis	Large and bright nucleated cells (*n* = 3, 11.1%)	Disorganized and irregular melanocytes scattered or nested distribution (*n* = 5, 18.5%)
	High‐refractive granules (*n* = 2, 7.4%)	Melanin granules (*n* = 4, 14.8%)

A diagnosis of ALM was made by histopathology (Figure 1B) in 27 cases. The 27 cases showed disordered epidermal structures. In 20 cases, upper transitional melanocytes of various sizes showing an irregular‐shape were observed. The melanocytes were characterized by an increased nuclear volume and intense nuclear staining. Intracellular melanin granules were present in 16 cases. In 27 cases, melanocytes were scattered or distributed in nests at the junction of the dermis and epidermis; the cells in the nests were of different sizes and showed intense nuclear staining and atypia. Scattered or nested melanocytes were observed in the dermis in five cases, showing large, darkly stained nuclei and atypical shapes; melanin granules were detected in the melanocytes in four cases. Two cases did not show melanoma features in the first pathological examination. However, RCM‐guided re‐biopsy led to a pathological diagnosis of ALM in the two cases. In addition to the 27 ALM cases, there were 2 cases of nevus (with active melanocyte growth), 1 case of basal cell carcinoma, and 1 case was diagnosed as seborrheic keratosis.

The results of RCM and histopathological examination were consistent in 29 of 31 patients (93.5%). Histopathology diagnosed 27 cases as ALM, 1 case as seborrheic keratosis, and 1 case as basal cell carcinoma. Two cases initially identified as ALM by RCM were subsequently confirmed to be pigmented nevi by histopathology. The results of RCM did not identify any false‐negative cases, although two false‐positive cases were later pathologically confirmed as acral nevi. The sensitivity of RCM for the diagnosis of ALM was 100%, specificity was 50%, positive predictive value was 93.1%, and negative predictive value was 100%.

## 4 DISCUSSION

Many studies have explored the RCM characteristics of MM,^1^, ^4^, ^5^ including melanoma in specific locations such as lentigo maligna melanoma.^6^, ^7^, ^8^ However, monitoring acral skin using RCM is challenging because of the thick stratum corneum of the extremities; there are few reports describing the RCM characteristics of acral melanoma. In this study, RCM showed a sensitivity of 100% for the diagnosis of ALM, which was higher than that of histopathology. In the horizontal growth phase of early ALM, histopathological examination detects freckle‐like proliferation of atypical melanocytes in the basal epidermal layer, which is characterized by increased size and intense nuclear staining of melanocytes. However, the limited biopsy material, which is common in ALM given its specific location, frequently leads to missed diagnoses. This can occur in cases in which certain lesions are not sampled or when atypical melanocytes are not observed. In this study, two patients were diagnosed with ALM by RCM. However, the initial biopsy did not identify tumor cells, and the patients were finally diagnosed by RCM‐guided re‐biopsy. The specificity of RCM in this study was only 50%, which was primarily due to the limited number of non‐ALM cases (four patients) among the selected subjects. The thick stratum corneum found on the palms and toes may prevent deep scanning by RCM, which may lead to missed diagnosis; however, the present results suggest that RCM has the capacity to detect lesions in these locations. In this study, the pathologically‐confirmed ALM cases showed characteristic RCM features, which may be attributed to both the thinning of the epidermis in ALM and the upward migration of melanoma tumor cells to the superficial granular layer or stratum corneum. In the fingers or toes, RCM detected deeper lesions, which may be due to the thinner skin in these areas. Cinotti et al.^9^, ^10^ detected highly refractive sweat gland duct openings in ALM. Although this feature is commonly detected in palm and toe skin lesions, it is not exclusive to ALM and was therefore not described in this study.

The primary diagnostic indicator for MM is the dispersion of round and oval Paget‐like cells in the epidermis, although spindle cells may be the most commonly detected cells in the early stages of ALM. The RCM analysis in this study showed that dendritic cells were predominant in 66.7% (18 out of 27) of ALM patients, as indicated by the presence of dark brown patches. Round or oval Paget cells were detected in 22.2% (6 cases) of patients. In skin lesions clinically characterized as plaque, RCM revealed infiltration by round and oval Paget‐like cells. The proliferation of dendritic melanocytes in the epidermis could mark the initial phase of melanoma growth. Subsequently, poorly differentiated tumor cells undergo clonal expansion, which results in a decrease in dendritic cells and the emergence of invasive round and oval cells. In the analysis of dendritic cells, RCM cannot distinguish between melanocytes and Langerhans cells. The presence of dendritic cells in >30% of lesions is a sensitive indicator for melanoma diagnosis.^11^ The appearance of round and oval cells at the dermal‐epidermal junction is another sensitive indicator for the diagnosis of ALM, irrespective of the amounts detected. The presence of large numbers of round cells at the dermal‐epidermal junction suggests the possibility of aggressive melanoma. In this study, RCM detected uneven highly refractive granules in the epidermis, a significant diagnostic clue for ALM, in 18 cases (66.7%). Natarelli et al.^12^ detected highly refractive pleomorphic cells, which were considered to be melanocytes, scattered in the stratum corneum at the positive edge of ALM by RCM, although the underlying epidermis appeared normal. This feature was also observed in three cases in the present study.

A strong correlation between RCM features and histopathological features was observed (Table 1). A disordered epidermis was evident in 27 patients, as determined by both RCM and histopathology; however, this feature is also present in other malignant tumors such basal cell carcinoma and squamous cell carcinoma. Regarding epidermal features, RCM showed good concordance with histopathology. Other features, such as the disappearance of epidermal processes at the dermal‐epidermal junction, structural disorder and nesting, atypical highly refractive cells, large and bright nucleated cells, and highly refractive granules in the dermis, were more frequently observed by histopathology than by RCM, which may be attributed to the limited depth of detection of RCM. Because of the specific location of acral nevi, there may be a small amount of Paget‐like spread or scattered atypical cells in the epidermis, leading to potential false‐positive results. In this study, two false‐positive cases diagnosed by RCM were pathologically confirmed to be acral nevi. Basal cell carcinoma and seborrheic keratosis were accurately diagnosed by RCM, thereby reducing the misdiagnosis of ALM. The present results suggest that RCM is a reliable and useful auxiliary tool for the diagnosis of ALM, which may decrease the need for histopathological biopsy to some extent.

RCM showed good agreement with histopathology in the diagnosis of ALM, thereby providing a reliable method for the non‐invasive diagnosis of ALM. However, this study had a limited sample size, and further studies with a larger sample size are therefore necessary.

## CONFLICT OF INTEREST STATEMENT

The authors declare no conflicts of interest.

## Data Availability

Research data are not shared.
